# N6-Methyladenosine Modification of PTTG3P Contributes to Colorectal Cancer Proliferation *via* YAP1

**DOI:** 10.3389/fonc.2021.669731

**Published:** 2021-09-30

**Authors:** Yang Zheng, Yue Wang, Yiyang Liu, Longfei Xie, Jinnian Ge, Guilin Yu, Guohua Zhao

**Affiliations:** ^1^ Department of Clinical Laboratory, Cancer Hospital of China Medical University, Liaoning Cancer Hospital & Institute, Liaoning, China; ^2^ Department of General Surgery, Cancer Hospital of China Medical University, Liaoning Cancer Hospital & Institute, Liaoning, China; ^3^ Department of Surgery, Affiliated Hospital of Youjiang Medical University for Nationalities, Guangxi, China; ^4^ Department of Physics and Integrative Biology, University of California, Berkeley, Berkeley, CA, United States; ^5^ Department of General Surgery, The Central Hospital of Shenyang Medical College, Liaoning, China

**Keywords:** proliferation, CRC, METTL3, IGF2BP2, YAP1

## Abstract

**Background:**

Long noncoding RNAs (lncRNAs) have emerged to have irreplaceable roles in the epigenetic regulation of cancer progression, but their biological functions in colorectal cancer (CRC) remain unclear.

**Methods:**

LncRNA expression profiles in CRC tissue and their normal counterpart were explored. Through gain and loss of function approaches, the role of lncRNA PTTG3P was validated in relevant CRC cells and subcutaneous tumor model. The correlations of PTTG3P expression with clinical outcomes were assessed.

**Results:**

PTTG3P was upregulated in CRC tissues and was closely correlated with unsatisfactory prognosis. PTTG3P facilitated glycolysis and proliferation, and the transcriptional regulator YAP1 was necessary for PTTG3P-induced proliferation. Mechanistically, the N6-methyladenosine (m6A) subunit METTL3 increased PTTG3P expression by influencing its stability, while insulin-like growth factor 2 mRNA binding protein 2 (IGF2BP2) could identify PTTG3P m6A methylation status and bind to it. IGF2BP2 knockdown partly recovered PTTG3P expression induced by METTL3, indicating that METTL3-regulated PTTG3P expression depended on the presence of IGF2BP2. Finally, rescue assays validated the critical role of the METTL3/PTTG3P/YAP1 axis on CRC proliferation.

**Conclusions:**

PTTG3P is an independent prognostic biomarker for CRC. The METTL3/PTTG3P/YAP1 axis promotes the progression of CRC and is a promising treatment target.

## Introduction

Colorectal cancer (CRC) remains a major cause of death from malignant tumors. As of 2012, CRC has become the second most common cancer in women (9.2% of cancer diagnoses) and the third most common cause in men (10.0%9) and is the fourth cause of cancer deaths after lung, stomach, and liver cancer (1, 2). Metabolic reprogramming in cancer is due to the oncogenic activation of signal transduction pathways and associated factors. Epigenetic mechanisms also contribute to regulating metabolic gene expression in cancer. Accumulating evidence suggests that metabolic alterations may affect the epigenome. Understanding the relationship between metabolism and epigenetics in cancer may provide new opportunities for anticancer treatment strategies (3).

Malignant tumor cells perform glycolysis at a rate that is 10 times faster than their noncancerous tissue counterparts (4). N6-methyladenosine (m6A) is responsible for the methylation of the nitrogen at position 6 of the adenosine base within mRNA and was first characterized in the 1970s (5). Currently, associations between m6A and malignant tumors have been reported in breast cancer, prostate cancer, pancreatic cancer, kidney cancer, leukemia, stomach cancer, and sarcoma (6–9). LncRNA PTTG3P, or pituitary tumor-transforming 3, pseudogene (PTTG3P) (accession no. NR_002734), is located at chromosome 8q13.1. It is an intronless gene that is highly homologous to its family members pituitary tumor-transforming 1 (PTTG1) and pituitary tumor-transforming 2 (PTTG2) and was first reported in the study of the human pituitary tumor transforming gene (hPTTG) family in 2000 (10).

Our study determined that METTL3 could increase PTTG3P expression, and highly expressed PTTG3P was predictive of unsatisfactory prognosis in patients with CRC. Further study revealed that PTTG3P facilitated proliferation by regulating the METTL3/PTTG3P/YAP1 axis. These findings may provide a rationale for PTTG3P as a potential therapeutic target for CRC treatment.

## Materials and Methods

### Clinical *S*amples

One hundred twenty patients with CRC were enrolled from the Affiliated Hospital of Youjiang Medical University for Nationalities, the Central Hospital of Shenyang Medical Hospital and Liaoning Cancer Hospital between March 2010 and November 2015. The including criteria were as follow: patients have definite pathological diagnosis and did not receive chemotherapy or radiotherapy before surgery. The tumor and paired non-tumor tissues were also collected after lesion excision within 30 min and stored in liquid nitrogen, then transferred to a −80°C refrigerator. The characteristics of cases were thoroughly noted. All of the CRC patients have signed informed consent before utilizing the clinical resources for investigation aims. The study was approved by the Ethics Committee of Youjiang Medical University for Nationalities and Liaoning Cancer Hospital.

### Cell Lines Culture

Five human CRC cell lines (HT29, SW620, HCT-8, SW480, and HCT-116) and normal human intestinal epithelial cell lines (FHC, NCM460) were obtained from ATCC (Manassas, VA, USA) and cultured according to their instructions. All cells were cultured in an incubator according to their instructions at 37°C and in a humidified atmosphere with 5% CO_2_.

### Total RNA Isolation, qRT−PCR, and Transfection

The expression levels of RNA were calculated by the quantitative real-time PCR (qRT-PCR) system. Total RNA was extracted by TRIzol Reagent (Invitrogen), and 1 μg of total RNA was reverse transcribed using the PrimeScript RT Reagent Kit (Perfect Real-Time; Takara). pcDNA3.1-PTTG3P and PTTG3P-containing lentiviral sequence vector (sh-PTTG3P) were purchased from GeneChem Corporation (Shanghai, China). CRC cells were transfected with plasmids in the presence of Lipofectamine 3000 (Invitrogen). After 48 h of transfection, cells were gathered for further use in the following experiments. The gene expression quantity was calculated using the 2^−ΔΔCt^ method. The detail is in **Supplementary Tables S1**, **S2**.

### Cell Proliferation Assay

Cell viability assay was carried out to analyze cell proliferation. Cell viability was estimated using CCK8 (CK04, DOJINDO, Beijing, China), on the basis of the manufacturer’s instruction. Cells were seeded in 96-well culture plates. After incubation for the indicated time, CCK-8 reagent (10 μl) was added to each well. Cell viability was measured with a microplate reader for absorbance at a wavelength of 450 nm.

### EdU Assay

The cells were incubated with 5-ethynyl-20-deoxyuridine (EdU) (Ribobio, Guangzhou, China) for 5 h and processed according to the manufacturer’s instruction. After three washes with phosphate buffer saline (PBS), the cells were treated with 200 μl of 1× Apollo^®^ reaction cocktail for 30 min. Then, the DNA contents of the cells in each well were stained with 100 μl of Hoechst 33342 (5 μg/ml) for 30 min and visualized under a fluorescence microscope.

### Flow Cytometry of Apoptosis

CRC cells in six-well plates were rinsed in PBS and then were trypsinized and resuspended in 100 μl binding buffer added with 2.5 μl of fluorescein isothiocyanate (FITC) conjugated Annexin V and 1 μl of PI (Invitrogen). Fifteen minutes later, flow cytometry (BD Biosciences) was utilized for apoptotic cells.

### Glucose, Lactate, Adenosine Triphosphate Levels, and Extracellular Acidification Rate

The levels of glucose and lactate were calculated with a Glucose Colorimetric Assay Kit (BioVision, CA) and a Lactate Assay Kit (BioVision, CA) in line with the instructions of the manufacturer. Adenosine triphosphate (ATP) level was tested using Cell Titer-Glo Luminescent Cell Viability Assay (Promega, Madison, MI). Extracellular acidification rate (ECAR) was detected using Seahorse XF 96 Extracellular Flux Analyzer (Agilent Technologies, Santa Clara, CA) according to the manufacturer’s instructions.

### m6A Analysis

The quantification of m6A RNA methylation level in total RNA was detected using the m6A RNA methylation detection kit (Epigentek, Farmingdale, NY), according to the manufacturer’s instructions.

### Methylated RNA Immune−Precipitation Assay

Total RNA was extracted from cells using TRIzol (Invitrogen) following the manufacturer’s instructions. m6A antibody (Abcam) and Magna methylated RNA immune-precipitation (MeRIP) m6AKit (Merck Millipore) were explored to immunoprecipitate chemically fragmented RNA (~100 nucleotides) according to its instruction. Enrichment of m6A containing RNA was measured by qRT-PCR.

### Animal *S*tudy

HCT-116 cells were transfected with sh-PTTG3P. Indicated cells (1 × 10^7^) were subcutaneously injected into 4-week-old male nude mice. Tumor volume was measured every 5 days. After 35 days, the mice were sacrificed, and the tumor weight was measured. The animal study was carried out following the Guide for the Care and Use of Laboratory Animals of the NIH. This study had been approved by the Committee on the Ethics of Animal Experiments of Youjiang Medical University for nationalities.

### Statistical *A*nalysis

All the data were shown as the mean ± standard deviation, in at least three independent experiments. The difference between the two independent groups was analyzed by a two-tailed Student’s t-test, while multigroup comparison was made by ANOVA. Expression correlation between genes was analyzed by Pearson correlation analysis. Survival analysis was conducted using the Kaplan–Meier method and analyzed by the log-rank test. SPSS 22.0 (SPSS Inc., Chicago, IL, USA) was used to conduct statistical analyses, and differences were ensured when *p*-value was <0.05.

## Results

### PTTG3P Was Highly Expressed in CRC

**Figure 1:**
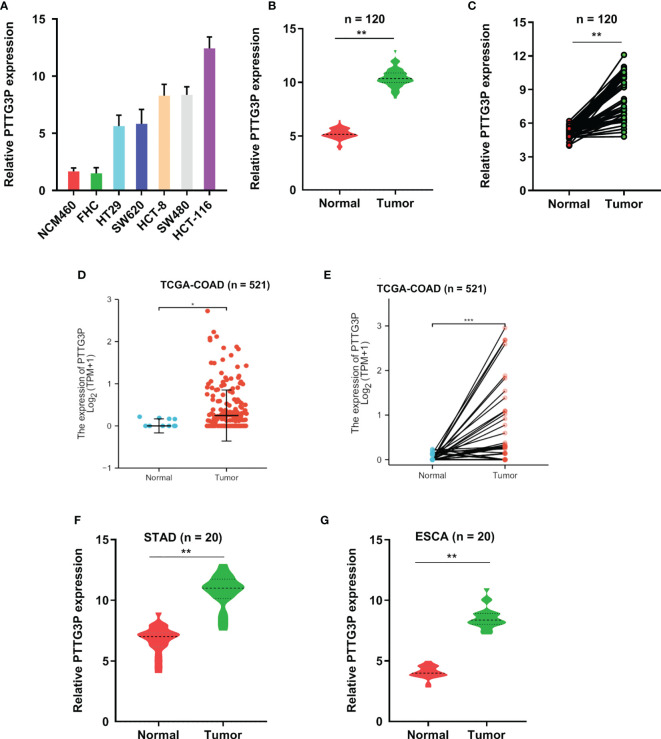
PTTG3P exhibits high expression in CRC. **(A)** The expression profiles of PTTG3P in NCM460, FHC, HT29, SW620, HCT-8, SW480 and HCT-116 were detected with qRT-PCR. **(B, C)** High PTTG3P expression was observed in 120 paired tumor and paired adjacent non-tumor tissues. **(D, E)** High PTTG3P expression was observed in the TCGA database of COAD (n=521). **(F, G)** High PTTG3P expression was observed in STAD (n=20) and ESCA (n=20) . *p < 0.05, **p < 0.01, ***p < 0.001.

To identify lncRNAs involved in CRC progression, we examined lncRNAs expression profiles using the GSE 84983 dataset (https://ftp.ncbi.nlm.nih.gov/geo/series/GSE84nnn/GSE84983/matrix/). We compared the gene expression between CRC tumor tissues and adjacent normal tissues; we focused on the upregulated lncRNAs (fold change >5, *p* < 0.01) in CRC tumor tissues, as these lncRNAs might potentially be identified oncogenes and therapeutic targets (**Supplementary Figure S1A**). The expression of LncRNA PTTG3P was significantly enhanced in CRC tumor tissues and thus became the focus of the present study (**Supplementary Figure S1B**). Through the analysis of the open-reading frames (ORFs) Finder and conserved domain database, we determined that PTTG3P could not consistently code proteins. Five other different online metrics confirmed the above conclusion (**Supplementary Tables S3**). No valid Kozak consensus sequence was identified in PTTG3P (11), indicating that PTTG3P is an lncRNA with no protein-coding potential. To verify the expression of PTTG3P in CRC, we investigated the detailed annotative process of preclinical human cancer models *via* the Cancer Cell Line Encyclopedia (CCLE) (www.broadinstitute.org/ccle) and found that PTTG3P was remarkably overexpressed in CRC cell lines (**Supplementary Figures 1E**, **F**). Next, the HT29, SW620, HCT-8, SW480, HCT-116, NCM460, and FHC cells were evaluated for PTTG3P expression. As shown in **Figure 1A**, PTTG3P expression was higher in HT29, SW620, HCT-8, SW480, and HCT-116 cells, compared with NCM460 and FHC cells. Furthermore, we explored PTTG3P expression in a cohort of 120 paired CRC and non-tumor tissues; the clinicopathological characteristics are reported in **Table 1**. PTTG3P was overexpressed in CRC tissues (**Figures 1B, C**), which was in accordance with the results of the findings using datasets from the Cancer Genome Atlas (TCGA) database (**Figures 1D, E**). In addition, high PTTG3P expression was observed in several types of malignant tumors (**Supplementary Figure S1G**). Furthermore, our specimens confirmed that PTTG3P was overexpressed in stomach adenocarcinoma (STAD) and esophageal squamous cell carcinoma (ESCA) (**Figures 1F, G**). Altogether, these data revealed that PTTG3P was elevated in CRC and might be an oncogene.

**Table 1:** Correlation between PTTG3P expression in serum and clinicopathologic characteristics of CRC patients.

Variable	PTTG3P expression			*p*-value
	Total (n = 120)	High expression	Low expression	
Age (years)				
≤60	52	27	26	0.86
>60	68	32	35	
Gender				
Male	56	30	28	0.74
Female	64	29	33	
Tumor size (cm)				
≤5	81	47	37	0.02
>5	39	16	24	
Tumor invasion depth				
T1–2	95	53	43	0.28
T3–4	25	12	20	
Lymph node metastasis				
N0	40	25	20	0.09
N1–2	80	36	39	
Vessel invasion				
Yes	65	49	20	0.06
No	55	20	31	
Differentiation				
Well	38	20	18	0.01
Moderate	62	46	16	
Poor	20	13	7	

### PTTG3P Correlated With Patient Prognosis

**Figure 2:**
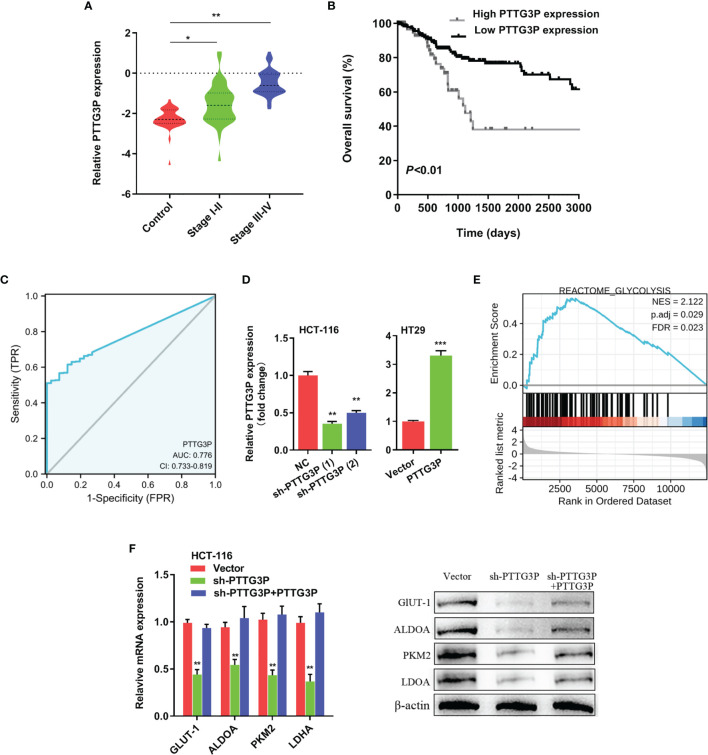
PTTG3P correlates with patient prognosis **(A)** PTTG3P expression was checked in different clinical stages of CRC tissues. **(B)** PTTG3P expression and survival predicted poor prognosis of overall survival in a cohort of 120 paired cases. **(C)** ROC curve analysis of the diagnostic ability of PTTG3P expression. **(D)** Short hairpin RNA (shRNA) targeting PTTG3P and PTTG3P overexpressed plasmids were transfected into HCT-116 and HT29 cells. **(E)** GSEA plot showing that PTTG3P expression positively correlated with glycolysis-activated gene signatures (REACTOME GLYCOLYSIS). **(F)** Analysis of glycolytic gene expression with PTTG3P knockdown or PTTG3P knockdown with PTTG3P re-expression. *p < 0.05, **p < 0.01, ***p < 0.001.

To identify the correlation between PTTG3P expression and clinicopathological features, we divided cases into low and high expression based on the median expression. Highly expressed PTTG3P was positively associated with tumor size (*p* = 0.02) and differentiation (*p* = 0.01), but not with age (*p* = 0.86), sex (*p* = 0.74), tumor invasion depth (*p* = 0.28), lymph node metastasis (*p* = 0.09), or vessel invasion (*p* = 0.06) (**Table 2**). PTTG3P was more highly expressed in stage III–IV (advanced stage) tumors than in stage I–II (early stage) tumors (**Figure 2A**). Kaplan–Meier survival curves revealed that patients with higher expression of PTTG3P had poorer survival (**Figure 2B**). Furthermore, we determined the prognostic ability of PTTG3P in CRC. As shown in **Table 2**, univariate analyses revealed that high expression of PTTG3P was associated with a dramatic risk of death (*p* < 0.01), and multivariate analysis showed that PTTG3P expression could be an independent prognostic factor (*p* < 0.01). Subsequently, receiver operating characteristic (ROC) curves was constructed to evaluate the diagnostic value of PTTG3P in CRC; the area under the ROC curve (AUC) was 0.776 [95% confidence interval (CI) 0.733–0.819] (**Figure 2C**). Thus, our findings suggested that higher expression of PTTG3P predicted a worse prognosis and may serve as an independent prognostic factor of disease outcome.

**Table 2:** Univariate and multivariate analyses of clinicopathologic characteristics for correlations with overall survival.

Variables	Univariate analysis		Multivariate analysis	
	HR (95%CI)	*p*-value	HR (95% CI)	*p*-value
PTTG3P expression	1.758 (1.085–2.850)	<0.01	1.712 (1.053–2.782)	<0.01
Tumor size	1.650 (1.086–2.508)	<0.01	1.923 (1.276–2.898)	<0.01
Differentiation	1.724 (1.183–2.511)	<0.01	1.724 (1.183–2.511)	<0.01

### Overexpression of PTTG3P Promoted CRC Cell Glycolysis and Proliferation

**Figure 3:**
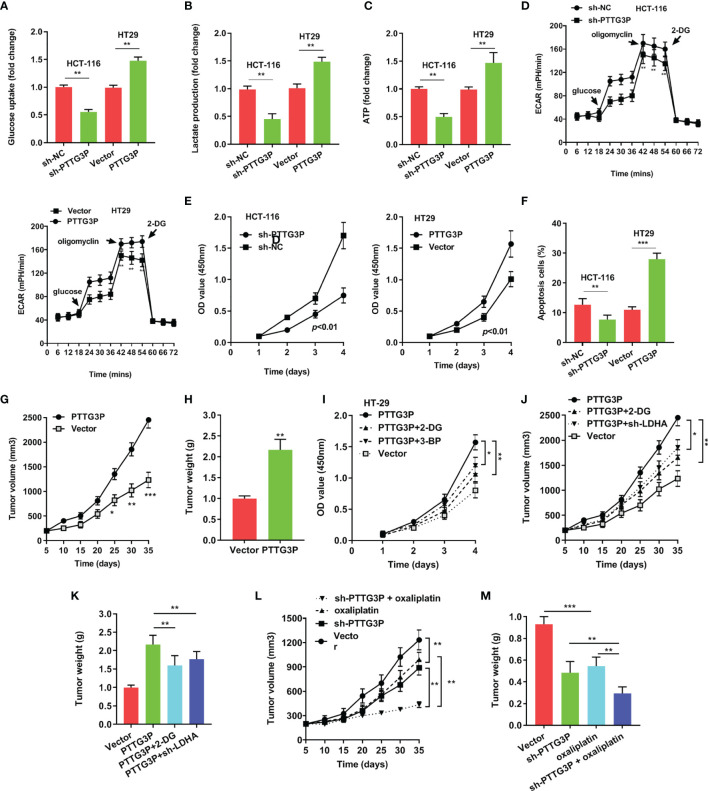
Overexpression of PTTG3P promoted CRC cell glycolysis and proliferation **(A)** Glucose uptake analysis, **(B)** Lactate production analysis, **(C)** ATP analysis explored with PTTG3P knockdown or PTTG3P overexpression in HCT116 or HT-29 cells. **(D)** Extracellular acidification rate (ECAR) analysis tested the glycolytic capacity with PTTG3P knockdown or PTTG3P overexpression in HCT116 or HT-29 cells. **(E)** CCK8 assay detected the proliferation of HCT-116 and HT29 cells transfected with PTTG3P knockdown or PTTG3P overexpression. **(F)** Flow cytometry assays revealed that PTTG3P affected cell apoptosis. **(G)** Tumor volume and **(H)** weight were measured in vivo when injected with overexpressed PTTG3P transfected HCT-116 cells. **(I)** CCK8 assay detected the proliferation of HT29 cells transfected with overexpressed PTTG3P and treated with 2.5mM 2-DG or 100 μM 3-BP. **(J)** Xenograft tumors volume, **(K)** Xenograft tumors weight was established, with injected with PTTG3P or PTTG3P plus sh-LDHA or PTTG3P treated with 2-DG (1000 mg/kg, injected into the abdominal cavity). **(L)** Tumor volume and **(M)** weight were measured in vivo when injected with PTTG3P knockdown (20 nmol twice per week) and oxaliplatin treatment (5 mg/kg twice per week, injected into the abdominal cavity) transfected HCT-116 cells. *p < 0.05, **p < 0.01, ***p < 0.001.

To explore the function of PTTG3P, we transfected PTTG3P-encoding plasmids and short hairpin RNA (shRNA)-targeting PTTG3P into HT29 and HCT-116 cells (**Figure 2D**). By determining PTTG3P expression *via* gene set enrichment analysis (GSEA) of TCGA profiles, we determined that PTTG3P expression was positively correlated with glycolysis and affected genes involved in glycolysis regulation (**Figure 2E**). Decreased PTTG3P levels were accompanied by depletion of GLUT-1, ALDOA, PKM2, and LDHA levels, which are regulator genes of glycolysis. These decreased gene expression could be rescued by re-expression of PTTG3P (**Figure 2F**). Next, we determined that PTTG3P depletion *in vitro* repressed glucose uptake, lactate production, ATP levels, and ECAR levels, whereas the opposite outcomes were observed after enforced expression of PTTG3P (**Figures 3A–D**). Furthermore, we carried out rescue experiments to explore whether GLUT-1, ALDOA, PKM2, and LDHA took part in PTTG3P regulation of glycolysis genes. We found that PTTG3P + si-GLUT-1 and PTTG3P + si-LDHA could partly rescue glucose uptake, PTTG3P + si-GLUT-1 could partly rescue lactate production, and PTTG3P + si-PKM2 could partly rescue glucose uptake (**Supplementary Figure S3**). Furthermore, we found that silenced PTTG3P expression suppressed cell proliferation and facilitated apoptosis, while PTTG3P overexpression enhanced these functions (**Figures 3E, F**). In the subcutaneous tumor model, PTTG3P overexpression facilitated tumor growth (**Figures 3G, H**). We then explored whether glycolysis played a vital role in proliferation. As expected, the glycolic inhibitors 2-DG and 3-BP or depletion of LDHA, which could catalyze the last step of glycolysis, partially abrogated cell proliferation and tumor growth (**Figures 3I, K**). In addition, EDU proliferation assays showed that the cell proliferation capacity of cancer cells with silenced PTTG3P expression was significantly lower compared to the control group. Furthermore, YAP1 could rescue the proliferation induced by PTTG3P depletion (**Supplementary Figure S4**).

Clinically, oxaliplatin is used for CRC treatment. Glycolysis suppression is an effective strategy for blocking cell proliferation and overcoming drug resistance (12). We speculated that PTTG3P ablation and oxaliplatin might play a synergistic antitumor effect. As shown in **Figures 3L, M**, PTTG3P depletion could be associated with simultaneous oxaliplatin treatment. Taken together, PTTG3P ablation plus oxaliplatin therapy was a promising strategy for treating CRC.

### YAP1 Depletion Partially Abrogated the Proliferation Induced by PTTG3P

**Figure 4:**
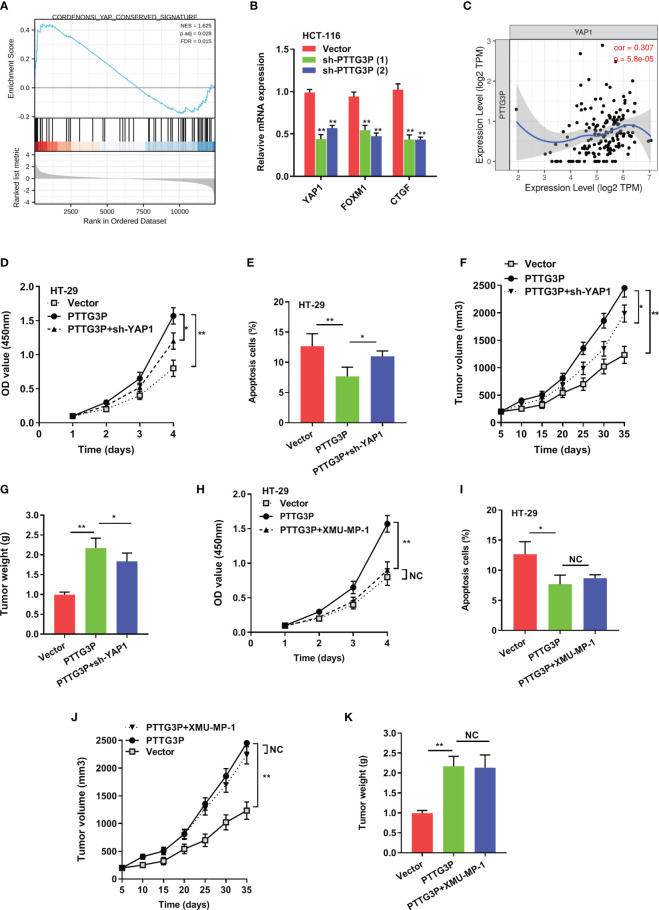
YAP1 depletion partially abrogated the proliferation caused by PTTG3P. **(A)** GSEA plot showing that PTTG3P expression positively correlated with YAP-activated gene signatures. **(B)** PTTG3P silencing impaired the mRNA level of YAP1, FOXM1 and CTGF. **(C)** The relationship between PTTG3P and YAP1 was analyzed by Spearman’s correlation analysis. **(D)** CCK8 assay showed that PTTG3P plus YAP1 knockdown partly rescued cell proliferation. **(E)** Flow cytometry assays revealed that PTTG3P plus YAP1 knockdown partly rescued cell apoptosis. **(F)** Xenograft tumors volume, **(G)** Xenograft tumors weight were established, with injected with PTTG3P or PTTG3P plus sh-YAP1. Empty vector as indicated. **(H)** CCK8 assay showed that PTTG3P plus XMU-MP-1barely rescued cell proliferation. **(I)** Flow cytometry assays revealed that PTTG3P plus XMU-MP-1barely rescued cell apoptosis. **(J)** Xenograft tumors volume, **(K)** Xenograft tumors weight was established, with injected with PTTG3P or PTTG3P plus XMU-MP-1. Data are presented as the mean ± SD from three independent experiments. *p < 0.05, **p < 0.01. NC, negative control.

To explain the pathways involved in PTTG3P-mediated CRC proliferation, GSEA using published TCGA colon adenocarcinoma (COAD) datasets were explored. Our analysis indicated that PTTG3P expression associated with Yes1-associated transcriptional regulator (YAP1) activated gene signatures, indicating that the Hippo signaling pathway might take part in PTTG3P function (**Figure 4A**). As verification of this speculation, hub genes in the Hippo pathway, including LATS1/2, MST1/2, and YAP1, and Hippo pathway target genes, such as CDX2, FOXM1, CTGF, and CYR61, were tested. We observed diminished PTTG3P interfered with the expression of YAP1, FOXM1, and CTGF (**Figure 4B**). YAP1 is a crucial factor in the Hippo pathway and is involved in cell proliferation and suppression of apoptotic genes. In this study, PTTG3P and YAP1 were positively associated (**Figure 4C**). Furthermore, YAP1 was highly expressed in tumor tissues in the TCGA datasets of COAD (**Supplementary Figures S1C, D**), and YAP1 was associated with advanced clinical characteristics of CRC (**Supplementary Table S4**). In addition, we designed and carried out a series of rescue experiments, PTTG3P plus YAP1 knockdown partly reversed proliferation, apoptosis, and tumor growth induced by PTTG3P (**Figures 4D–G**); however, treatment with the Hippo pathway inhibitor, XMU-MP-1 (an inhibitor of MST1/2), barely induced any effects on these processes (**Figures 4H–K**). This suggests that PTTG3P might bypass the key factor MST1/2 while modulating YAP1 to display pivotal functions.

### m6A Modification Was Involved in PTTG3P Expression

**Figure 5:**
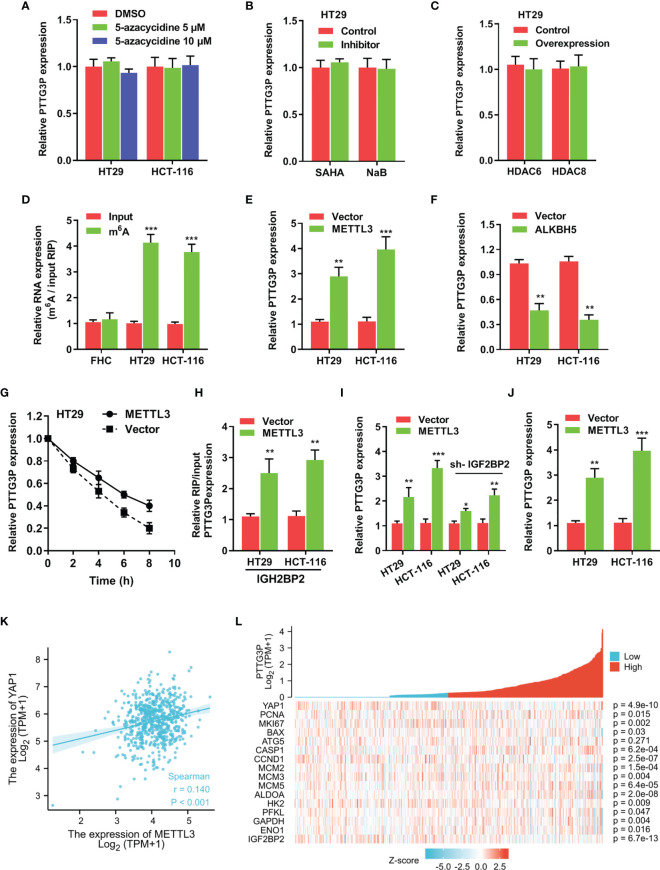
m6A modification is involved in the ectopic expression of PTTG3P in CRC **(A)** qRT-PCR analysis of PTTG3P treated with DMSO or 5-Azacytidine (5 μM or 10 μM) for 72 hr (n = 3). **(B)** HT29 cells were treated with SAHA (2 µM), or NaB (2 mM) for 24 h, and PTTG3P expression was measured. **(C)** After transfection with vector control, pcDNA/HDAC6, or pcDNA/HDAC8 for 24 h, PTTG3P expression in HT29 cells was measured by qRT-PCR. **(D)** MeRIP-qPCR showed the m6A modification expression in FHC cells as compared with the HT29 and HCT-116 cells. **(E)** The qRT-PCR analysis of PTTG3P levels in control and METTL3 overexpression in HT29 and HCT-116 cells. **(F)** The qRT-PCR analysis of PTTG3P levels in control and ALKBH5 overexpression in HT29 and HCT-116 cells. **(G)** RNA stability analysis showed the stability of PTTG3P in HT29 cells treated with actinomycin D (Act-D, 5 μg/m). **(H)** After transfection with vector or METTL3 for 24 h, the binding of PTTG3P and IGF2BP2 was analyzed by RIP-PCR in HT29 and HCT-116 cells. **(I)** After transfection with IGF2BP2 knockdown, the PTTG3P level increased by METTL3 was partly rescued. **(J)** METTL3 increased the level of YAP1 analyzed by qRT-PCR in HT-29 and HCT116 cells. **(K)** METTL3 and YAP1 are positively correlated from the TCGA database of colon adenocarcinoma (COAD). **(L)** PTTG3P co-expression heat map, TCGA (https://portal.gdc.cancer.gov/) COAD, level 3 HTSeq FPKM. Data are presented as the mean ± SD from three independent experiments. *p < 0.05, **p < 0.01, ***p < 0.001.

To determine specific factors involved in regulating PTTG3P expression, we applied DNA methyltransferase inhibition in HT29 and HCT-116 cells, and no influence was found on PTTG3P expression (**Figure 5A**). Next, we exposed these cell lines to SAHA and NaB, broad-spectrum HDAC inhibitors, to examine whether histone acetylation exerted a role in PTTG3P expression, and discovered that HDAC inhibitors failed to alter PTTG3P levels (**Figure 5B**). Neither HDAC6 nor HDAC8 influenced PTTG3P expression (**Figure 5C**). Subsequently, MeRIP-qPCR indicated that m6A modification was dramatically increased in HT29 and HCT-116 cells (**Figure 5D**). The methylation of adenosine is directed by a large m6A methyltransferase complex containing METTL3 as the SAM-binding subunit. We confirmed that METTL3 significantly increased the level of PTTG3P expression (**Figure 5E**). Fat mass and obesity-associated protein (FTO) and demethylase alkB homolog 5 (ALKBH5) have been described as m6A demethylases (13, 14). Next, we determined that ALKBH5 suppressed PTTG3P expression (**Figure 5F**). Next, we conducted RNA stability analyses by treating cells with Act-D, which binds DNA at the initiation complex and prevents RNA chain elongation. We found that METTL3 strengthened the stability of PTTG3P (**Figure 5G**). The biological functions of m6A are mediated through a group of RNA binding proteins that specifically recognize the methylated adenosine on RNA. Recently, insulin-like growth factor-2 mRNA-binding proteins 1, 2, and 3 (IGF2BP1-3) have been described as m6A readers. We performed RNA immunoprecipitation PCR (RIP-PCR) to evaluate the potential binding of IGF2BP1-3 to PTTG3P. The results indicated that IGF2BP2 could bind to PTTG3P, and METTL3 strengthened their binding (**Figure 5H**). Interestingly, IGF2BP2 knockdown could partly rescue the PTTG3P expression increased by METTL3 (**Figure 5I**). METTL3 could increase the expression level of YAP1 and was positively correlated with YAP1 expression (**Figures 5J–L**). Finally, the association between METTL3 and IGF2BP2 expression and clinicopathological characteristics from TCGA are summarized in **Supplementary Tables S5**, **S6**.

### The METTL3/PTTG3P/YAP1 Axis Was Vital for CRC Proliferation

**Figure 6:**
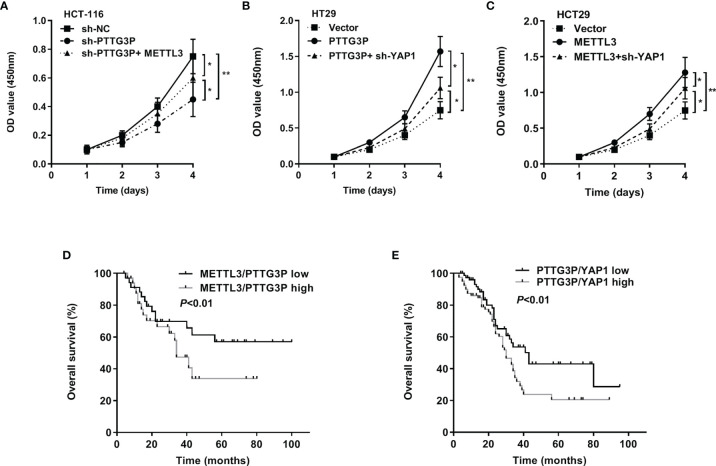
The METTL3/PTTG3P/YAP1 axis is vital for CRC progression **(A)** CCK8 assay detected the proliferation of HCT-116 cells transfected with sh-PTTG3P or sh-PTTG3P+METTL3. **(B)** CCK8 assay detected the proliferation of HT29 cells transfected with PTTG3P or PTTG3P+sh-YAP1. **(C)** CCK8 assay detected the proliferation of HCT-116 cells transfected with METTL3 or METTL3sh-YAP1. **(D)** Kaplan-Meier analysis of the OS curves for patients with METTL3/PTTG3P-high (both levels of METTL3/PTTG3P were high), METTL3/PTTG3P-low (both levels of METTL3/PTTG3P were low) expression. **(E)** Kaplan-Meier analysis of the OS curves for patients with PTTG3P/YAP1-high (both levels of PTTG3P/YAP1 were high), PTTG3P/YAP1-low (both levels of PTTG3P/YAP1 were low) expression. Data are presented as the mean ± SD from three independent experiments. *p < 0.05, **p < 0.01.

To evaluate the involvement of the METTL3/PTTG3P/YAP1 axis in CRC proliferation, we carried out a series of rescue experiments in HCT-116 and HT29 cells and found that PTTG3P KD plus METTL3, PTTG3P plus YAP1 KD, and METTL3 plus YAP1 KD could partly recover the proliferative phenotype (**Figures 6A–C**). Hence, the METTL3/PTTG3P/YAP1 axis played a pivotal role in CRC progression. Clinically, the METTL3/PTTG3P high and PTTG3P/YAP1 high groups defined a more unsatisfactory prognosis than low group (**Figures 6D, E**). Furthermore, higher levels of METTL3, ALKBH5, and IGF2BP2 predicted poor prognosis and diagnostic value in CRC using the TCGA dataset (**Supplementary Figure S2**).

## Discussion

Pseudogenes may be transcribed into RNA at low levels due to promoter elements inherited from the ancestral gene or arising by new mutations. Although most transcripts have rarely been reported to have functional significance, other than chance transcripts from other parts of the genome, some pseudogenes have given rise to regulatory RNAs and new proteins. For instance, the lncRNA HK2P1, a pseudogene of HK2, promoted lactate production and glucose uptake in endometrial stromal cells (15). Pseudogene PTENP1 repressed the oncogenic PI3K/AKT pathway and inhibited hepatocellular carcinoma (HCC) progression (16). Our findings provide evidence that PTTG3P facilitates CRC progression *via* the METTL3/PTTG3P/YAP1 axis, and PTTG3P has a potential diagnostic value, with an AUC of 0.776 (95% CI, 0.733–0.819). Clinically, high PTTG3P expression significantly associates with tumor size and TNM stage and shorter survival time. Furthermore, our data are in line with other research studies. Liu et al. (17) reported that PTTG3P is markedly upregulated in CRC tissues. Zhou et al. (18) proposed that PTTG3P is a valuable marker of HCC progression and is useful for biomarker development. Weng et al. (19) reported that PTTG3P facilitates cell proliferation, migration, invasion, and serves as a new promising strategy for interfering with gastric cancer. In addition, PTTG3P plays an important role in breast cancer (20) and pancreatic cancer (21). Thus, the oncogenic role of PTTG3P is strongly supported by findings in the literature.

Malignant tumors undergo glycolysis at a higher speed than normal tissue (22, 23). This phenomenon is known as the Warburg effect, which demonstrates that a malignant tumor is caused by mitochondrial metabolism disorder (24). Doherty et al. (23) found that tumor lactate levels correlate with increased metastasis, tumor recurrence, and poor outcomes. Thus, targeting lactate metabolism is a prospective method for cancer therapeutics. Furthermore, cancer cells with a high level of glycolysis and acid resistance have an energetic growth advantage, which facilitates unrestrained proliferation and invasion. In this study, we proposed that PTTG3P could increase glycolysis by regulating genes linked with metabolic pathways. Recently, the ketogenic diet was used to constrain glycolysis to starve cancer cells, by adjusting mitochondrial metabolism (25). The Hippo signaling pathway has become increasingly important in human cancer (26); the key regulator YAP1 is upregulated in breast cancer, colorectal cancer, and liver cancer (27); and YAP1 promotes proliferation (28–30) and inhibits apoptosis (30). Clinically, YAP1 is used as a target for cancer drug development (31). Yi et al. (32) suggested that inhibiting TEAD–YAP1 interactions or blocking the binding function of WW domains is a pharmacologically viable strategy against the activity of the YAP1 oncoprotein. We discovered that PTTG3P activates the Hippo signaling pathway by promoting YAP1, FOXM1, and CTGF, but not MST1/2 expression. The impact of m6A on cancer cell proliferation might be much more profound. The depletion of METTL3 is known to cause apoptosis of cancer cells and may reduce their invasiveness (33, 34), while the activation of ALKBH5 by hypoxia has been shown to induce cancer stem cell enrichment (35). Our data demonstrated that METTL3 and ALKBH5 coordinately mediated the m6A modification of PTTG3P expression, whereas IGF2BP2 mediated m6A-dependent functions; that is, METTL3-enhanced PTTG3P expression depended on IGF2BP2 activity.

To investigate whether PTTG3P might regulate the expression of the genes such as YAP1, GLUT-1, ALDOA, PKM2, and LDHA *via* a competing endogenous RNA (ceRNA) mechanism or directly by binding to a common motif, we used the database ENCORI to analyze the relationship between PTTG3P and those genes. However, neither the ceRNA mechanism nor direct binding showed regulation of PTTG3P and YAP1, GLUT-1, ALDOA, PKM2, and LDHA. In the future, we will carry out RNA pulldown and mass spectrometry to identify proteins directly binding to PTTG3P or RIP-qPCR to identify YAP1, GLUT-1, ALDOA, PKM2, and LDHA binding RNA.

Overall, our study revealed the METTL3/PTTG3P/YAP1 axis in CRC progression, and m6A readers IGF2BP2 takes part in this progress. Hence, PTTG3P might be a useful target for CRC prevention and therapy and may shed some light on the role of the poorly understood m6A and pseudogene in cancer biology.

## Data Availability Statement

The datasets used and analyzed in the current study are available from the corresponding author on reasonable request.

## Ethics Statement

The studies involving human participants were reviewed and approved by Ethics committee of Liaoning Cancer Hospital and Affiliated Hospital of Youjiang Medical University for Nationalities. The patients/participants provided their written informed consent to participate in this study. The animal study was reviewed and approved by China medical university. Written informed consent was obtained from the individual(s) for the publication of any potentially identifiable images or data included in this article.

## Author Contributions

The work presented here was carried out in collaboration between all authors. YW and GZ contributed to the conception of the study. YZ and YL contributed significantly to analysis and manuscript preparation. GY and JG performed the data analyses and wrote the manuscript. LX helped perform the analysis with constructive discussions. All authors contributed to the article and approved the submitted version.

## Funding

This work was supported by the Natural Science Foundation of Liaoning Province of China (grant numbers 20180550778 and 20180551043) and National Natural Science Cultivation Foundation of China of Liaoning Cancer Hospital (grant numbers 2021-ZLLH-18 and 2020-ZLLH-48).

## Conflict of Interest

The authors declare that the research was conducted in the absence of any commercial or financial relationships that could be construed as a potential conflict of interest.

## Publisher’s Note

All claims expressed in this article are solely those of the authors and do not necessarily represent those of their affiliated organizations, or those of the publisher, the editors and the reviewers. Any product that may be evaluated in this article, or claim that may be made by its manufacturer, is not guaranteed or endorsed by the publisher.

## Supplementary Material

Supplementary Figure S1
**(A)** According to an online database (GSE84983), the volcano graph showed differential gene. **(B)** qRT-PCR of the expressions of the top 5 up-regulated lncRNAs in CRC. **(C, D)** High YAP1 expression was observed in CRC (TCGA-COAD, n = 521). **(E, F)** Exploring PTTG3P expression in CRC cell lines by assembling the Cancer Cell Line Encyclopedia (CCLE) (www.broadinstitute.org/ccle). **(G)** High PTTG3P expression was observed in many kinds of tumors. ***P < 0.001.

Supplementary Figure S2
**(A)** ROC curve of PTTG3P, METTL3, YAP1 and IGF2BP2. **(B)** Survival curve of METTL3 in progress free interval. **(C)** Survival curve of ALKBH5 in overall survival, disease specific survival and progress free interval. **(D)** Survival curve of IGF2BP2 in overall survival, disease specific survival and progress free interval.

Supplementary Figure S3
**(A)** Rescue assay was performed, PTTG3P+si-GLUT1 and PTTG3P+si-LDHA could abrogate the PTTG3P induced glucose uptake. **(B)** Rescue assay was performed, PTTG3P+si-GLUT1 could abrogate the PTTG3P induced lactate production. **(C)** Rescue assay was performed, PTTG3P+si-PKM2 could abrogate the PTTG3P induced ATP accumulation. *P < 0.05, **P < 0.01.

Supplementary Figure S4 The cancer cells proliferation capacity was detected by EdU assays in HCT-116 cell lines transfected with the sh-NC, sh-PTTG3P and sh-PTTG3P+YAP1 plasmid (Scale bar, 20μm).
