# Supplementary figures and images for: N6-Methyladenosine Modification of PTTG3P Contributes to Colorectal Cancer Proliferation via YAP1
Source: Front Oncol. 2021 Sep 30;11:669731. doi: 10.3389/fonc.2021.669731 (PMC8515845; doi:10.3389/fonc.2021.669731)

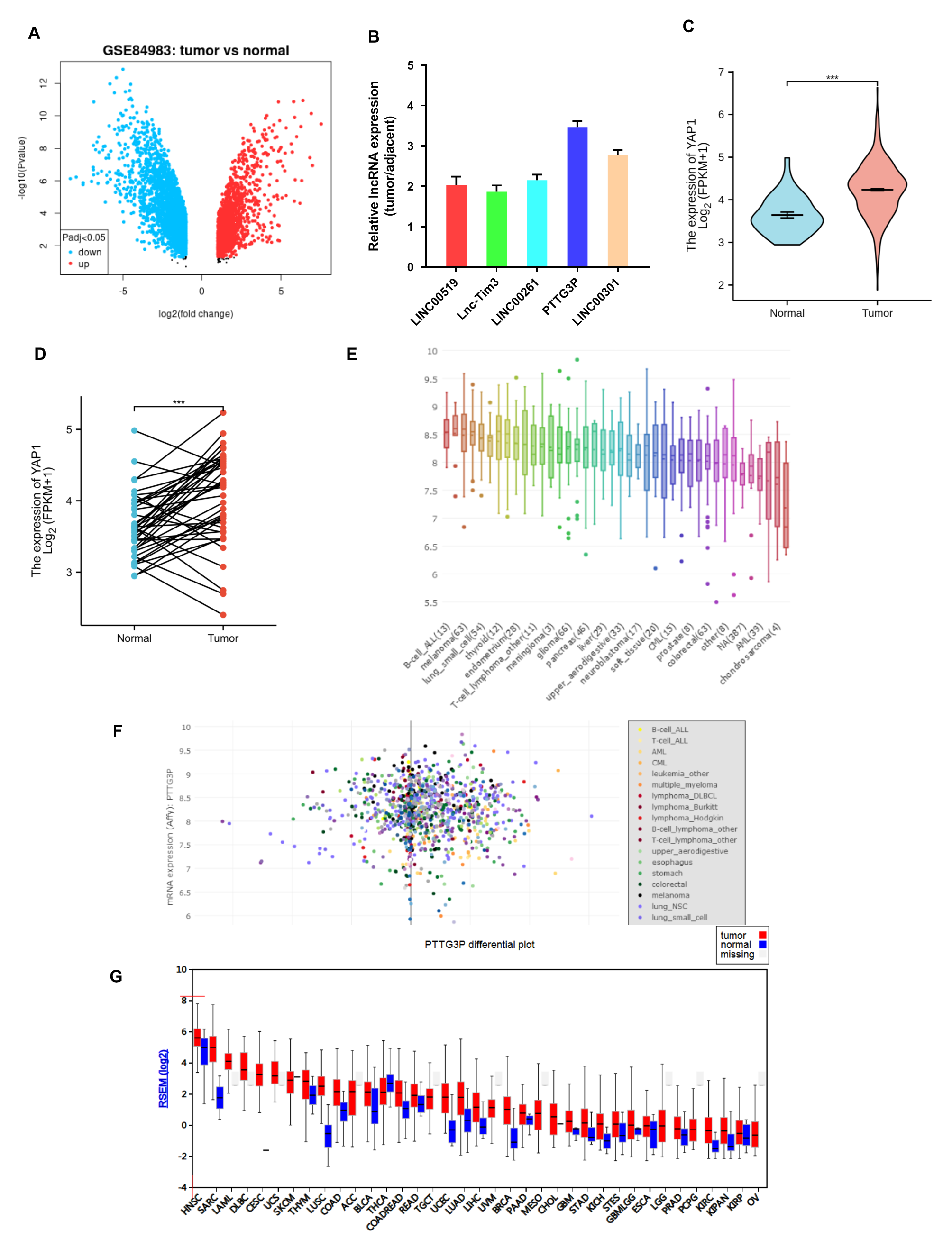

Supplement: Supplementary Figure S1 — (A) According to an online database (GSE84983), the volcano graph showed differential gene. (B) qRT-PCR of the expressions of the top 5 up-regulated lncRNAs in CRC. (C, D) High YAP1 expression was observed in CRC (TCGA-COAD, n = 521). (E, F) Exploring PTTG3P expression in CRC cell lines by assembling the Cancer Cell Line Encyclopedia (CCLE) (www.broadinstitute.org/ccle). (G) High PTTG3P expression was observed in many kinds of tumors. ***P < 0.001. [file Image_1.tif]

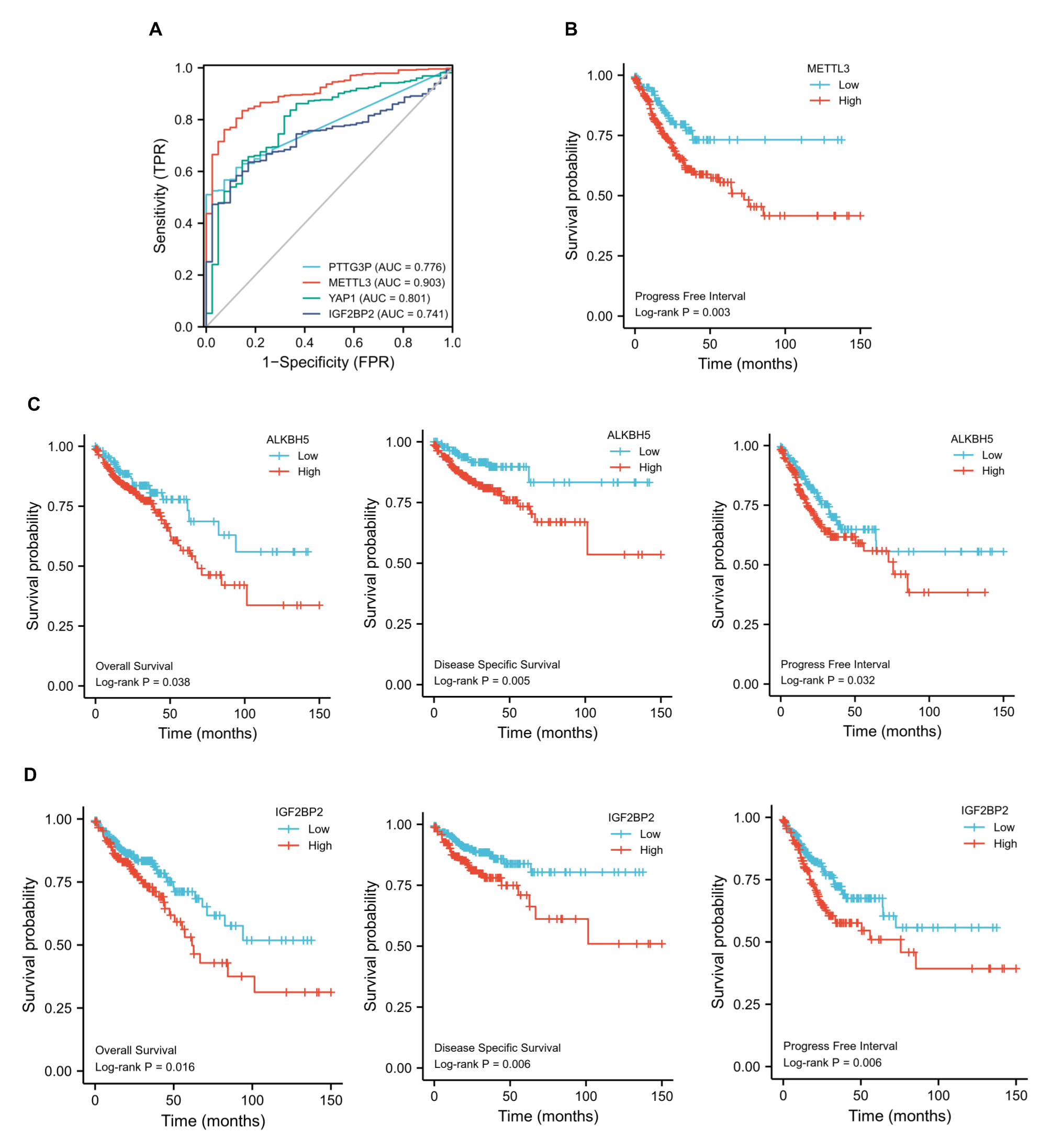

Supplement: Supplementary Figure S2 — (A) ROC curve of PTTG3P, METTL3, YAP1 and IGF2BP2. (B) Survival curve of METTL3 in progress free interval. (C) Survival curve of ALKBH5 in overall survival, disease specific survival and progress free interval. (D) Survival curve of IGF2BP2 in overall survival, disease specific survival and progress free interval. [file Image_2.tif]

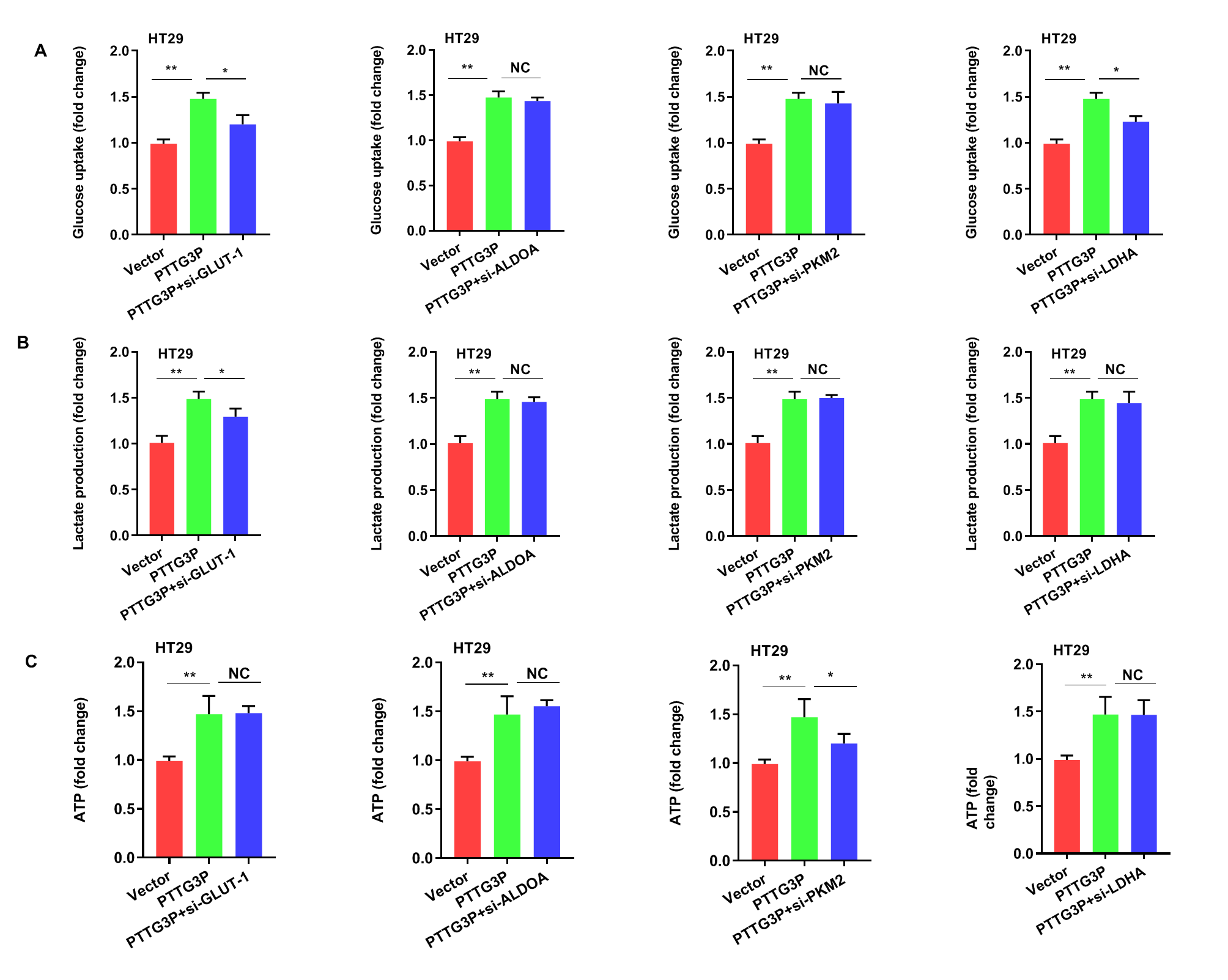

Supplement: Supplementary Figure S3 — (A) Rescue assay was performed, PTTG3P+si-GLUT1 and PTTG3P+si-LDHA could abrogate the PTTG3P induced glucose uptake. (B) Rescue assay was performed, PTTG3P+si-GLUT1 could abrogate the PTTG3P induced lactate production. (C) Rescue assay was performed, PTTG3P+si-PKM2 could abrogate the PTTG3P induced ATP accumulation. *P < 0.05, **P < 0.01. [file Image_3.tif]

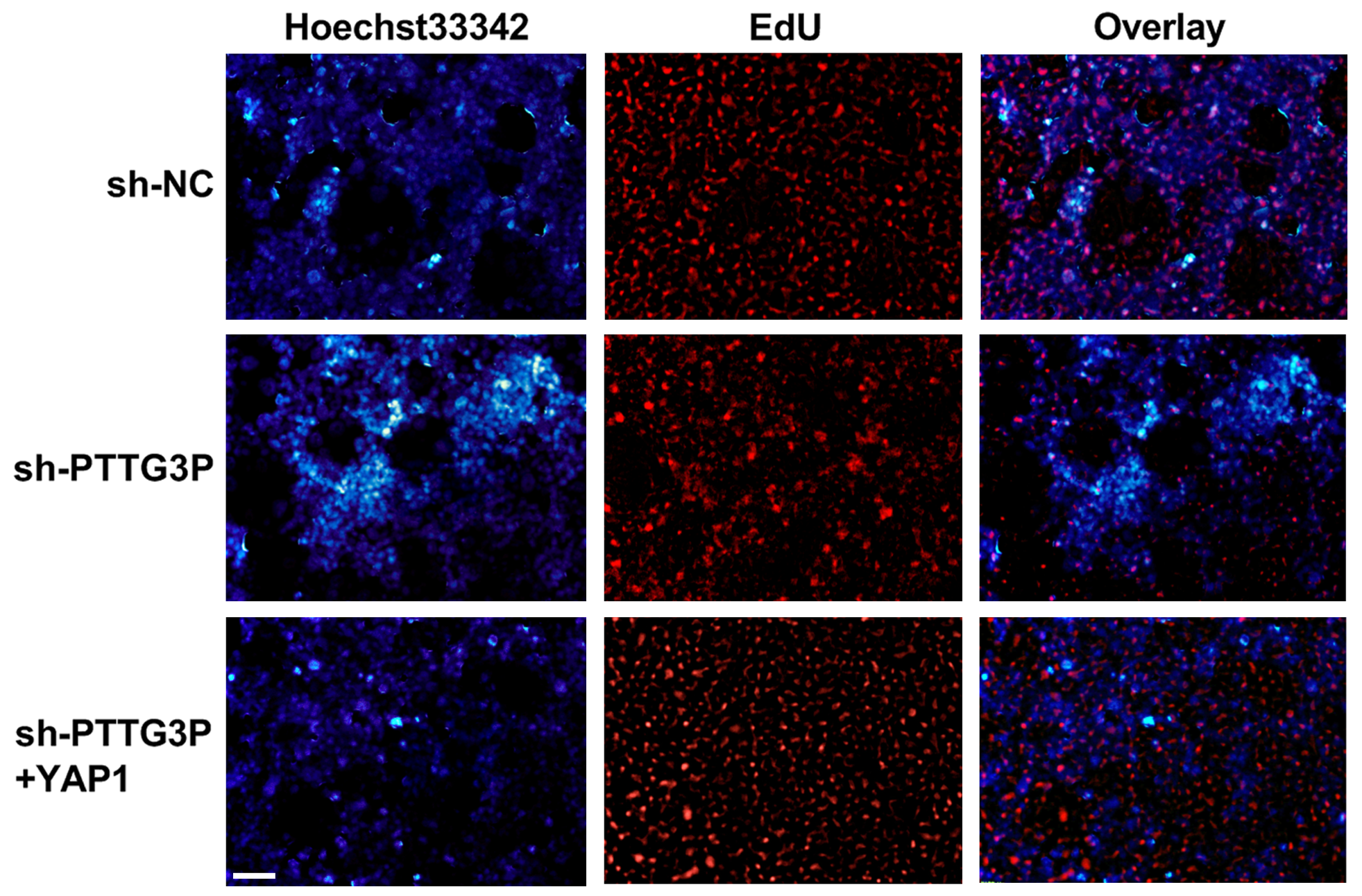

Supplement: Supplementary Figure S4 — The cancer cells proliferation capacity was detected by EdU assays in HCT-116 cell lines transfected with the sh-NC, sh-PTTG3P and sh-PTTG3P+YAP1 plasmid (Scale bar, 20μm). [file Image_4.tif]
