# Supplementary material for: N6-Methyladenosine Modification of PTTG3P Contributes to Colorectal Cancer Proliferation via YAP1
Source: Front Oncol. 2021 Sep 30;11:669731. doi: 10.3389/fonc.2021.669731 (PMC8515845; doi:10.3389/fonc.2021.669731)
Supplement: Supplementary file 5 [file DataSheet_1.doc]

**Supplementary Table 1. sh-RNA sequences.**

| shRNA | Target sequence |
| --- | --- |
| sh-PTTG3P | GGTTGAGAGCGGCAATAATCC |
| sh-PTTG3P | GCATCCTTGTGGCTACAAAGG |
| sh-LDHA | GTTCACAAGCAGGTGGTTGAGAGTGCTTA |
| sh-PGK1 | ACAACCAGAGGATTAAGGC |
| sh-YAP1(1) | GGUCAGAGAUACUUCUUAAAU |
| sh-YAP1(2) | GGUGAUACUAUCAACCAAAGC |

**Supplementary Table 2. All primers for real-time PCR assay**

| Primer | Former | Reverse |
| --- | --- | --- |
| PTTG3P | GGGGTCTGGACCTTCAATCAA | GCTTTAGGTAAGGATGTGGGA |
| LDHA | ATGGCAACTCTAAAGGATCA | GCAACTTGCAGTTCGGGC |
| IGF2BP2 | GCCCCTCATTAAGCCCAAG | TTGTGGTGGTCTGACAGTTCG |
| GLUT-1 | GGCCAAGAGTGTGCTAAAGAA | ACAGCGTTGATGCCAGACAG |
| PKM2 | ATGTCGAAGCCCCATAGTGAA | TGGGTGGTGAATCAATGTCCA |
| ALDOA | AGGCCATGCTTGCACTCAGAAGT | AGGGCCCAGGGCTTCAGCAGG |
| CTGF | CAGCATGGACGTTCGTCTG | CAGCATGGACGTTCGTCTG |
| CYR61 | CTCGCCTTAGTCGTCACCC | CTCGCCTTAGTCGTCACCC |
| U6 | GATTTCTCCCTCATCGCTTACAG | CTGCTTCATGATCGTTGTTGCTTG |
| GAPDH | GGGAGCCAAAAGGGTCATCA | TGATGGCATGGACTGTGGTC |

**Supplementary Table 3. Protein coding potential**

| Metric | Raw result | interpretation |
| --- | --- | --- |
| PRIDE reprocessing 2.0 | 0 | Non-coding |
| Lee translation initiation sites | 0 | Non-coding |
| PhyloCSF score | <0 | Non-coding |
| CPAT coding probability | 2.33% | Non-coding |
| Bazzini small ORFs | 0 | Non-coding |

**Supplementary Table 4. Correlation between YAP1 expression and clinicopathologic characteristics of CRC patients**

| Characteristic | Low expression of YAP1 | High expression of YAP1 | p |
| --- | --- | --- | --- |
| n | 239 | 239 |  |
| T stage, n (%) |  |  | 0.756 |
| T1 | 7 (1.5%) | 4 (0.8%) |  |
| T2 | 39 (8.2%) | 44 (9.2%) |  |
| T3 | 161 (33.8%) | 162 (34%) |  |
| T4 | 31 (6.5%) | 29 (6.1%) |  |
| N stage, n (%) |  |  | 0.235 |
| N0 | 151 (31.6%) | 133 (27.8%) |  |
| N1 | 50 (10.5%) | 58 (12.1%) |  |
| N2 | 38 (7.9%) | 48 (10%) |  |
| M stage, n (%) |  |  | 0.046 |
| M0 | 182 (43.9%) | 167 (40.2%) |  |
| M1 | 25 (6%) | 41 (9.9%) |  |
| Pathologic stage, n (%) |  |  | 0.086 |
| Stage I | 40 (8.6%) | 41 (8.8%) |  |
| Stage II | 104 (22.3%) | 83 (17.8%) |  |
| Stage III | 63 (13.5%) | 70 (15%) |  |
| Stage IV | 25 (5.4%) | 41 (8.8%) |  |
| Primary therapy outcome, n (%) |  |  | 0.466 |
| PD | 9 (3.6%) | 16 (6.4%) |  |
| SD | 1 (0.4%) | 3 (1.2%) |  |
| PR | 6 (2.4%) | 7 (2.8%) |  |
| CR | 104 (41.6%) | 104 (41.6%) |  |
| Gender, n (%) |  |  | 0.169 |
| Female | 121 (25.3%) | 105 (22%) |  |
| Male | 118 (24.7%) | 134 (28%) |  |
| Race, n (%) |  |  | 0.457 |
| Asian | 4 (1.3%) | 7 (2.3%) |  |
| Black or African American | 23 (7.5%) | 40 (13.1%) |  |
| White | 104 (34%) | 128 (41.8%) |  |
| Weight, n (%) |  |  | 0.768 |
| <=90 | 84 (30.8%) | 105 (38.5%) |  |
| >90 | 35 (12.8%) | 49 (17.9%) |  |
| Age, n (%) |  |  | 0.226 |
| <=65 | 90 (18.8%) | 104 (21.8%) |  |
| >65 | 149 (31.2%) | 135 (28.2%) |  |
| Height, n (%) |  |  | 0.134 |
| <170 | 61 (23.8%) | 66 (25.8%) |  |
| >=170 | 49 (19.1%) | 80 (31.2%) |  |
| BMI, n (%) |  |  | 0.814 |
| <25 | 36 (14.1%) | 51 (19.9%) |  |
| >=25 | 74 (28.9%) | 95 (37.1%) |  |
| Residual tumor, n (%) |  |  | 0.430 |
| R0 | 185 (49.5%) | 161 (43%) |  |
| R1 | 1 (0.3%) | 3 (0.8%) |  |
| R2 | 11 (2.9%) | 13 (3.5%) |  |
| CEA level, n (%) |  |  | 0.404 |
| <=5 | 101 (33.3%) | 95 (31.4%) |  |
| >5 | 49 (16.2%) | 58 (19.1%) |  |
| Perineural invasion, n (%) |  |  | 0.329 |
| NO | 57 (31.5%) | 78 (43.1%) |  |
| YES | 15 (8.3%) | 31 (17.1%) |  |
| Lymphatic invasion, n (%) |  |  | 0.569 |
| NO | 129 (29.7%) | 137 (31.6%) |  |
| YES | 87 (20%) | 81 (18.7%) |  |
| History of colon polyps, n (%) |  |  | 0.038 |
| NO | 123 (30.1%) | 139 (34.1%) |  |
| YES | 85 (20.8%) | 61 (15%) |  |
| Colon polyps present, n (%) |  |  | 0.965 |
| NO | 67 (26.9%) | 95 (38.2%) |  |
| YES | 37 (14.9%) | 50 (20.1%) |  |
| Neoplasm type, n (%) |  |  | 1.000 |
| Colon adenocarcinoma | 239 (50%) | 239 (50%) |  |
| Rectum adenocarcinoma | 0 (0%) | 0 (0%) |  |
| OS event, n (%) |  |  | 0.373 |
| Alive | 192 (40.2%) | 183 (38.3%) |  |
| Dead | 47 (9.8%) | 56 (11.7%) |  |
| DSS event, n (%) |  |  | 0.385 |
| Alive | 201 (43.5%) | 197 (42.6%) |  |
| Dead | 28 (6.1%) | 36 (7.8%) |  |
| PFI event, n (%) |  |  | 0.121 |
| Alive | 183 (38.3%) | 167 (34.9%) |  |
| Dead | 56 (11.7%) | 72 (15.1%) |  |
| Age, meidan (IQR) | 69 (60, 77) | 68 (57, 77) | 0.287 |

**Supplementary Table 5. Correlation between METTL3 expression and clinicopathologic characteristics of CRC patients**

| Characteristic | Low expression of METTL3 | High expression of METTL3 | p |
| --- | --- | --- | --- |
| n | 239 | 239 |  |
| T stage, n (%) |  |  | 0.324 |
| T1 | 7 (1.5%) | 4 (0.8%) |  |
| T2 | 41 (8.6%) | 42 (8.8%) |  |
| T3 | 166 (34.8%) | 157 (32.9%) |  |
| T4 | 24 (5%) | 36 (7.5%) |  |
| N stage, n (%) |  |  | 0.039 |
| N0 | 137 (28.7%) | 147 (30.8%) |  |
| N1 | 65 (13.6%) | 43 (9%) |  |
| N2 | 37 (7.7%) | 49 (10.3%) |  |
| M stage, n (%) |  |  | 1.000 |
| M0 | 179 (43.1%) | 170 (41%) |  |
| M1 | 34 (8.2%) | 32 (7.7%) |  |
| Pathologic stage, n (%) |  |  | 0.921 |
| Stage I | 39 (8.4%) | 42 (9%) |  |
| Stage II | 91 (19.5%) | 96 (20.6%) |  |
| Stage III | 69 (14.8%) | 64 (13.7%) |  |
| Stage IV | 34 (7.3%) | 32 (6.9%) |  |
| Primary therapy outcome, n (%) |  |  | 0.261 |
| PD | 9 (3.6%) | 16 (6.4%) |  |
| SD | 2 (0.8%) | 2 (0.8%) |  |
| PR | 5 (2%) | 8 (3.2%) |  |
| CR | 113 (45.2%) | 95 (38%) |  |
| Gender, n (%) |  |  | 0.410 |
| Female | 108 (22.6%) | 118 (24.7%) |  |
| Male | 131 (27.4%) | 121 (25.3%) |  |
| Race, n (%) |  |  | 0.296 |
| Asian | 7 (2.3%) | 4 (1.3%) |  |
| Black or African American | 26 (8.5%) | 37 (12.1%) |  |
| White | 115 (37.6%) | 117 (38.2%) |  |
| Weight, n (%) |  |  | 1.000 |
| <=90 | 91 (33.3%) | 98 (35.9%) |  |
| >90 | 40 (14.7%) | 44 (16.1%) |  |
| Age, n (%) |  |  | 0.641 |
| <=65 | 94 (19.7%) | 100 (20.9%) |  |
| >65 | 145 (30.3%) | 139 (29.1%) |  |
| Height, n (%) |  |  | 0.060 |
| <170 | 54 (21.1%) | 73 (28.5%) |  |
| >=170 | 71 (27.7%) | 58 (22.7%) |  |
| BMI, n (%) |  |  | 0.788 |
| <25 | 44 (17.2%) | 43 (16.8%) |  |
| >=25 | 81 (31.6%) | 88 (34.4%) |  |
| Residual tumor, n (%) |  |  | 0.732 |
| R0 | 168 (44.9%) | 178 (47.6%) |  |
| R1 | 2 (0.5%) | 2 (0.5%) |  |
| R2 | 14 (3.7%) | 10 (2.7%) |  |
| CEA level, n (%) |  |  | 0.134 |
| <=5 | 107 (35.3%) | 89 (29.4%) |  |
| >5 | 48 (15.8%) | 59 (19.5%) |  |
| Perineural invasion, n (%) |  |  | 0.249 |
| NO | 64 (35.4%) | 71 (39.2%) |  |
| YES | 27 (14.9%) | 19 (10.5%) |  |
| Lymphatic invasion, n (%) |  |  | 0.677 |
|  |  |  |  |
| NO | 131 (30.2%) | 135 (31.1%) |  |
| YES | 87 (20%) | 81 (18.7%) |  |
| History of colon polyps, n (%) |  |  | 0.205 |
| NO | 125 (30.6%) | 137 (33.6%) |  |
| YES | 80 (19.6%) | 66 (16.2%) |  |
| Colon polyps present, n (%) |  |  | 1.000 |
| NO | 79 (31.7%) | 83 (33.3%) |  |
| YES | 42 (16.9%) | 45 (18.1%) |  |
| Neoplasm type, n (%) |  |  | 1.000 |
| Colon adenocarcinoma | 239 (50%) | 239 (50%) |  |
| Rectum adenocarcinoma | 0 (0%) | 0 (0%) |  |
| OS event, n (%) |  |  | 0.656 |
| Alive | 190 (39.7%) | 185 (38.7%) |  |
| Dead | 49 (10.3%) | 54 (11.3%) |  |
| DSS event, n (%) |  |  | 0.863 |
| Alive | 201 (43.5%) | 197 (42.6%) |  |
| Dead | 31 (6.7%) | 33 (7.1%) |  |
| PFI event, n (%) |  |  | 0.121 |
| Alive | 183 (38.3%) | 167 (34.9%) |  |
| Dead | 56 (11.7%) | 72 (15.1%) |  |
| Age, meidan (IQR) | 69 (59, 77) | 68 (57.5, 78) | 0.578 |

**Supplementary Table 6. Correlation between IGF2BP2 expression and clinicopathologic characteristics of CRC patients**

| Characteristic | Low expression of IGF2BP2 | High expression of IGF2BP2 | p |
| --- | --- | --- | --- |
| n | 239 | 239 |  |
| T stage, n (%) |  |  | 0.982 |
| T1 | 6 (1.3%) | 5 (1%) |  |
| T2 | 42 (8.8%) | 41 (8.6%) |  |
| T3 | 162 (34%) | 161 (33.8%) |  |
| T4 | 29 (6.1%) | 31 (6.5%) |  |
| N stage, n (%) |  |  | 0.208 |
| N0 | 151 (31.6%) | 133 (27.8%) |  |
| N1 | 47 (9.8%) | 61 (12.8%) |  |
| N2 | 41 (8.6%) | 45 (9.4%) |  |
| M stage, n (%) |  |  | 0.070 |
| M0 | 183 (44.1%) | 166 (40%) |  |
| M1 | 26 (6.3%) | 40 (9.6%) |  |
| Pathologic stage, n (%) |  |  | 0.166 |
| Stage I | 44 (9.4%) | 37 (7.9%) |  |
| Stage II | 102 (21.8%) | 85 (18.2%) |  |
| Stage III | 65 (13.9%) | 68 (14.6%) |  |
| Stage IV | 26 (5.6%) | 40 (8.6%) |  |
| Primary therapy outcome, n (%) |  |  | 0.663 |
| PD | 12 (4.8%) | 13 (5.2%) |  |
| SD | 2 (0.8%) | 2 (0.8%) |  |
| PR | 5 (2%) | 8 (3.2%) |  |
| CR | 113 (45.2%) | 95 (38%) |  |
| Gender, n (%) |  |  | 0.783 |
| Female | 111 (23.2%) | 115 (24.1%) |  |
| Male | 128 (26.8%) | 124 (25.9%) |  |
| Race, n (%) |  |  | 0.734 |
| Asian | 3 (1%) | 8 (2.6%) |  |
| Black or African American | 26 (8.5%) | 37 (12.1%) |  |
| White | 95 (31%) | 137 (44.8%) |  |
| Weight, n (%) |  |  | 0.735 |
| <=90 | 80 (29.3%) | 109 (39.9%) |  |
| >90 | 33 (12.1%) | 51 (18.7%) |  |
| Age, n (%) |  |  | 0.514 |
| <=65 | 93 (19.5%) | 101 (21.1%) |  |
| >65 | 146 (30.5%) | 138 (28.9%) |  |
| Height, n (%) |  |  | 1.000 |
| <170 | 53 (20.7%) | 74 (28.9%) |  |
| >=170 | 53 (20.7%) | 76 (29.7%) |  |
| BMI, n (%) |  |  | 0.230 |
| <25 | 41 (16%) | 46 (18%) |  |
| >=25 | 65 (25.4%) | 104 (40.6%) |  |
| Residual tumor, n (%) |  |  | 0.496 |
| R0 | 188 (50.3%) | 158 (42.2%) |  |
| R1 | 1 (0.3%) | 3 (0.8%) |  |
| R2 | 12 (3.2%) | 12 (3.2%) |  |
| CEA level, n (%) |  |  | 0.533 |
| <=5 | 92 (30.4%) | 104 (34.3%) |  |
| >5 | 55 (18.2%) | 52 (17.2%) |  |
| Perineural invasion, n (%) |  |  | 0.540 |
| NO | 47 (26%) | 88 (48.6%) |  |
| YES | 19 (10.5%) | 27 (14.9%) |  |
| Lymphatic invasion, n (%) |  |  | 0.802 |
| NO | 130 (30%) | 136 (31.3%) |  |
| YES | 85 (19.6%) | 83 (19.1%) |  |
| History of colon polyps, n (%) |  |  | 0.001 |
| NO | 117 (28.7%) | 145 (35.5%) |  |
| YES | 90 (22.1%) | 56 (13.7%) |  |
| Colon polyps present, n (%) |  |  | 0.743 |
| NO | 64 (25.7%) | 98 (39.4%) |  |
| YES | 37 (14.9%) | 50 (20.1%) |  |
| Neoplasm type, n (%) |  |  | 1.000 |
| Colon adenocarcinoma | 239 (50%) | 239 (50%) |  |
| Rectum adenocarcinoma | 0 (0%) | 0 (0%) |  |
| OS event, n (%) |  |  | 0.373 |
| Alive | 192 (40.2%) | 183 (38.3%) |  |
| Dead | 47 (9.8%) | 56 (11.7%) |  |
| DSS event, n (%) |  |  | 0.212 |
| Alive | 205 (44.4%) | 193 (41.8%) |  |
| Dead | 27 (5.8%) | 37 (8%) |  |
| PFI event, n (%) |  |  | 0.179 |
| Alive | 182 (38.1%) | 168 (35.1%) |  |
| Dead | 57 (11.9%) | 71 (14.9%) |  |
| Age, meidan (IQR) | 71 (58.5, 78) | 68 (58, 77) | 0.170 |
